# An Optically Transparent Water‐Based Metamaterial Absorber for Ultra‐Broadband EMI Shielding in Coal Mines

**DOI:** 10.1002/advs.202518619

**Published:** 2026-01-18

**Authors:** Xiaojun Huang, Lina Gao, Yidan Xu, Yifei Wang, Wei Hou, Yu Luo

**Affiliations:** ^1^ College of Communication and Information Engineering Xi'an University of Science and Technology Xi'an China; ^2^ Engineering Research Center of Smart Coal Mine Advanced Communication Technology Universities of Shaanxi Province Xi'an China; ^3^ Daliuta Coal Mine Shendong Coal Group China Energy Investment Corporation Shenmu Shaanxi China; ^4^ National Key Laboratory of Microwave Photonics College of Electronic and Information Engineering Nanjing University of Aeronautics and Astronautics Nanjing China

**Keywords:** absorbers, ITO films, transparency, ultra‐wideband, water‐based

## Abstract

Electromagnetic interference (EMI) in underground coal mines, generated by large‐scale machinery and electronic devices, severely disrupts the operation of sensitive electronics, leading to performance degradation, equipment failure, and significant safety hazards. To address these challenges, we propose an ultra‐wideband, ultra‐thin, and optically transparent metamaterial absorber (MA) that combines patterned indium tin oxide (ITO) films with a water‐filled resin shell. This design, independent of the incident polarization maintains high absorption efficiency over a wide range of incident angles. The absorber achieves over 90% microwave absorption efficiency across an ultra‐wide frequency ranging from 0.52 to 40 GHz, corresponding to a remarkable relative bandwidth of 194.9%, with a thickness of only 1/50 of the maximum operating wavelength. Experimental evaluations conducted in a simulated coal mine tunnel environment demonstrate its exceptional EMI shielding performance: the MA effectively stabilized digital tube displays that previously flickered under strong interference and restored normal operation to analog multimeters exhibiting erratic behavior. These results confirm the capability of our absorber to enhance the operational reliability and measurement accuracy of sensitive electronic equipment in high‐EMI conditions while preserving optical transparency for real‐time visual monitoring. The proposed MA offers a promising solution for robust electromagnetic protection in complex and harsh electromagnetic environments.

## Graphical abstract

This study introduces a novel metamaterial absorber integrating water as a lossy medium with patterned indium tin oxide (ITO) films to achieve simultaneous optical transparency and ultra‐wideband electromagnetic absorption over 0.52‐40 GHz. It effectively overcomes the limitations of conventional opaque and bulky shielding materials, offering a transformative solution for electromagnetic interference protection in coal mines.

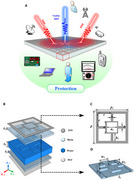

## 1 Introduction

The underground environment of coal mines presents one of the most challenging electromagnetic compatibility (EMC) scenarios for modern electronic systems. The extensive use of high‐power electromechanical equipment—such as mining machinery, frequency converters, and ventilation systems—generates intense and complex electromagnetic interference (EMI) [1]. This interference severely disrupts the operation of sensing instruments, communication devices, and control systems, leading to measurement inaccuracies, operational failures, and even critical safety hazards. For instance, EMI can cause erroneous readings in gas detectors or interrupt wireless communications, directly jeopardizing production safety and miners’ lives [2].

Conventional EMI shielding approaches, such as metallic enclosures, are often ineffective in this context. While they provide attenuation, they are opaque, bulky, and prone to corrosion in humid and dusty conditions. Moreover, they prevent visual monitoring of instrument status, which is essential for real‐time decision‐making in underground operations. The emergence of metamaterial has opened new pathways for EMI shielding [3, 4, 5]. These artificially engineered structures have attracted significant attention due to their exceptional capability to manipulate electromagnetic waves [6, 7]. Their customizable nature enables diverse applications, including but not limited to imaging [8, 9], cloaking [10, 11], sensing [12, 13, 14], modulation [15, 16, 17], and energy harvesting [18, 19]. Typically constructed from laminated metal‐dielectric composites [20, 21, 22], metamaterials can also be engineered to achieve high absorption across selected bands [23, 24, 25, 26, 27, 28]. However, most traditional metamaterial absorbers (MAs) remain optically opaque, non‐flexible, and ill‐suited to the environmental and operational constraints of coal mining [29].

Recent advances in fluid‐based metamaterials have highlighted the unique advantages of water as a tunable and lossy medium [30]. Its fluidity facilitates flexible metamaterial design [31, 32, 33, 34], while solute doping enables absorption tunability [35, 36, 37] Furthermore, its inherent polarity confers high dielectric loss and frequency dispersion, making it exceptionally effective for high‐frequency EM absorption [38, 39, 40, 41, 42]. Nevertheless, water‐based MAs alone often suffer from limited low‐frequency (<1 GHz) performance—a critical drawback in mining environments where low‐frequency EMI is prevalent [43, 44, 45]. Moreover, according to Rozanov's theorem, achieving low‐frequency absorption usually requires increased thickness [46], contradicting the need for compact and lightweight shielding solutions. Furthermore, the electromagnetic environment in the coal mine is not static. The operational frequencies of communication systems may need to change, and different types of mining equipment can introduce time‐varying EMI sources. Therefore, an ideal EMI shielding solution would not only be transparent and broadband but also possess a certain degree of frequency agility or tunability to adapt to these dynamic operational requirements, paving the way for next‐generation intelligent mining systems.

To overcome these challenges, we propose a metamaterial absorber that strategically integrates a patterned indium tin oxide (ITO) film with a water‐filled resin framework. In this design, the patterned ITO layer acts as the primary component, responsible for generating the fundamental electrical resonance and dominating the absorption performance, particularly at lower frequencies. Meanwhile, the water filler serves as a crucial functional medium that enhances the overall performance by improving impedance matching and strengthening dielectric loss mechanisms, which is essential for achieving ultra‐broadband absorption at higher frequencies. This synergistic combination has enabled the development of an ultra‐wideband, optically transparent, and ultra‐thin MA that operates effectively in the harsh conditions of coal mines. This design not only overcomes the conventional trade‐offs among bandwidth, thickness, and transparency, but also introduces a corrosion‐resistant and field‐deployable shielding solution. The proposed absorber exhibits polarization‐insensitive and wide‐angle absorption, achieving over 90% absorptivity from 0.52 to 40 GHz—a relative bandwidth of 194.9%—with a thickness of only 1/50 of the maximum operating wavelength. More importantly, its optical transparency allows continuous visual access to instrument panels and displays, a critical feature for real‐time monitoring and operational safety. Through experimental validation in a simulated mining environment, we demonstrate that our MA can effectively stabilize sensitive electronics such as digital displays and analog meters under strong EMI.

This work underscores a significant step forward in EMC management for smart mining and industrial IoT applications. By offering robust electromagnetic protection without sacrificing visibility or adaptability, the proposed MA holds great promise for enhancing the reliability and safety of electronic systems in complex electromagnetic environments such as coal mines.

## 2 Results and Discussion

### 2.1 Structure Design

**FIGURE 1:**
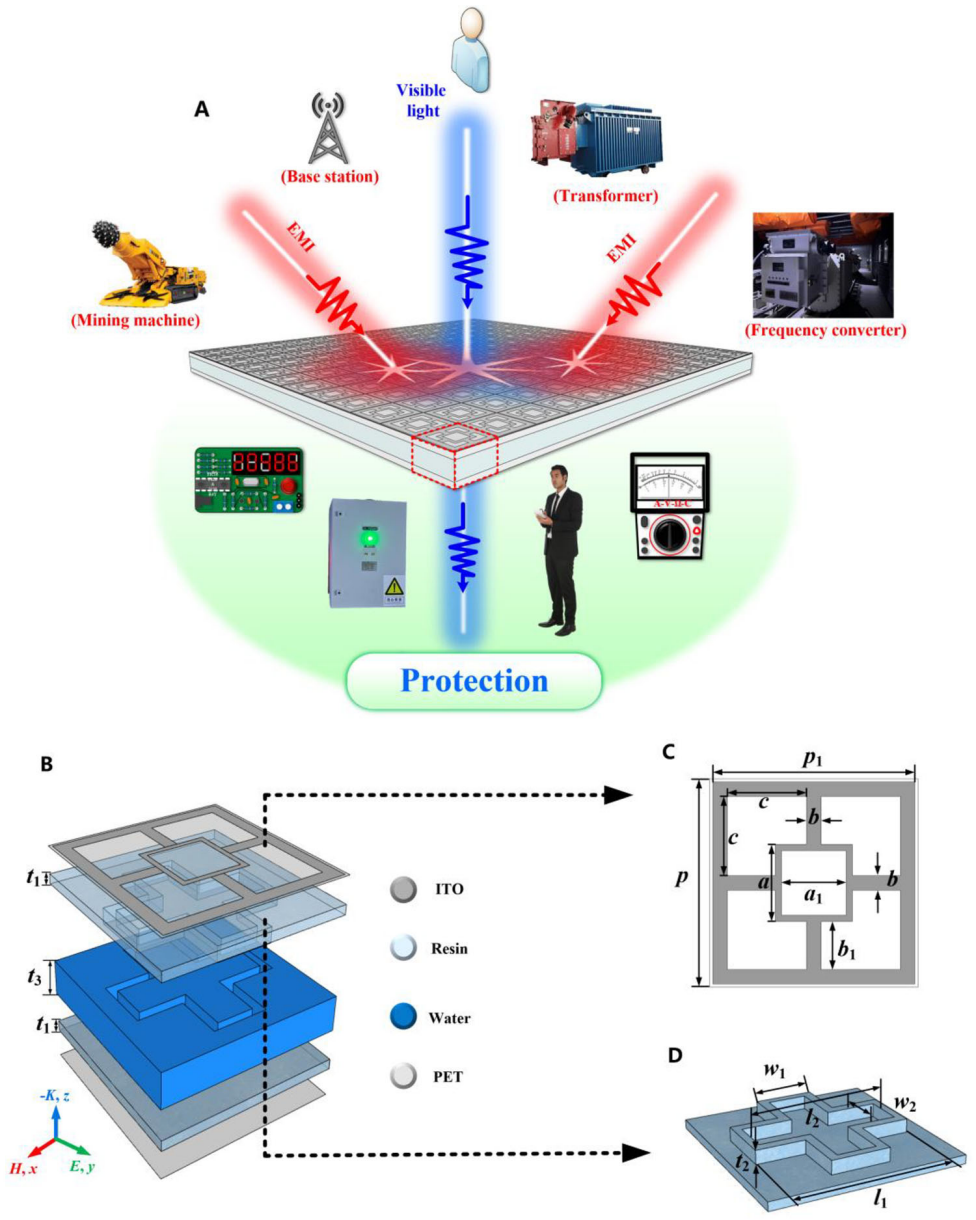
Geometry and design parameters of water‐based optically transparent MA: (A) overall schematic for electromagnetic protection; (B) unit cell side view; (C) top‐view layout of the ITO resonant layer; (D) the middle cross‐patterned resin layer.

The proposed optically transparent, water‐based MA is depicted in Figure 1a. Each unit cell consists of four functional layers: a high‐square‐resistance indium tin oxide (ITO) resonant layer, a structured resin layer, a water layer, and a low‐square‐resistance ITO reflective backplane. To enhance mechanical stability, the ITO layers are deposited on flexible polyethylene terephthalate (PET) substrates. The sheet resistances of the top ITO resonant layer and the bottom ITO reflective layer are denoted as R_1_ and R_2_, respectively. The resin layer features a specially designed cross‐patterned cavity, which, upon water injection, forms a well‐defined water absorption region. This multi‐layer architecture exploits constructive interference and impedance matching mechanisms to effectively dissipate incident EM waves. The complex permittivity of water is modeled using the Debye equation under standard conditions (23°C, atmospheric pressure). The transparent resin material exhibits a complex dielectric constant of 4.0(1 ‐ j0.001), and the PET substrate has a relative permittivity of 3.1(1 ‐ j0.03).

ITO is an optically transparent conductive oxide film. High‐quality ITO films can achieve a visible light transmittance exceeding 90% [47], which is the key to enabling the optical transparency of our proposed absorber. Its frequency‐dependent complex permittivity, ε, is derived from a modified Drude free electron model (see Section S1) [47]. PET substrates are adopted for their excellent stability, thermal resistance, and flexibility. The top PET layer is coated with a patterned high‐sheet‐resistance ITO film, while the bottom layer is fully covered with an unpatterned low‐sheet‐resistance ITO film, both fabricated via laser etching. Water is selected as the lossy medium due to its strong dielectric loss characteristics, accurately captured by the Debye relaxation model (see Section S2) [48]. These material properties collectively enable the structure's high absorption and transparency.

As shown in Figure 1c, the top ITO pattern consists of an outer square ring (side length *p*_1_), an inner square ring (dimensions *a* × *a*_1_), and four connecting rectangular strips (*b*, *b*_1_). The resin layer, depicted in Figure 1d, is comprised of a solid base with a cross‐shaped inlay, with thicknesses of *t*_1_ and *t*_2_, respectively. A side view of the unit cell (Figure 1b) indicates that the maximum height of the injected water layer is *t*_2_+*t*_3_. The total thickness of the PET layers is *t*_5_. The ITO layer itself is nanometer scale and is modeled as an infinitesimal thickness *t*_0_. Simulations were performed using CST EM simulation software, with the *x* and *y* directions defined as cell boundary conditions and the *z* direction set as open boundary conditions. Following optimization, the parameter values were determined as follows: *p *= 45 mm, *p*_1_ = 44 mm, *a *= 18 mm, *a*_1_ = 15 mm, *b *= 5 mm, *b*_1_ = 8.5 mm, *c *= 15 mm, *t*_0_ = 0.0001 mm, *t*_1_ = *t*_3_ = *t*_4_ = 3 mm, *t*_2_ = 4 mm, *t*_5_ = 0.185 mm, *l*_1_ = 38 mm, *w*_1_ = 14 mm, *l*_2_ = 36 mm, *w*_2_ = 10 mm, *R*_1_ = 800 Ω/sq, *R*_2_ = 8 Ω/sq.

When the EM waves impinge perpendicularly, the absorption of the absorber can be calculated using the equation *A*(*ω*) = 1‐*R*(*ω*)‐*T*(*ω*) = 1‐|*S*_11_|^2^‐|*S*_21_|^2^, where *R*(*ω*) and *T*(*ω*) represent reflectivity, and transmissivity, respectively, while *S*_11_ and *S*_21_ denote reflection and transmission coefficients, respectively. The high‐square resistance ITO film on the top layer enhances resonance with the incident EM waves, achieving effective impedance matching. Simultaneously, the low‐sheet‐resistance ITO film on the bottom layer acts as a reflective barrier, preventing the transmission of absorbed EM waves. When transmissivity is zero, indicating that the bottom layer functions as a highly conductive surface, the absorption can be simplified to *A*(*ω*) = 1‐*R*(*ω*) = 1‐|*S*_11_|^2^. These design features collectively ensure optimal absorption performance across the targeted frequency range.

**FIGURE 2:**
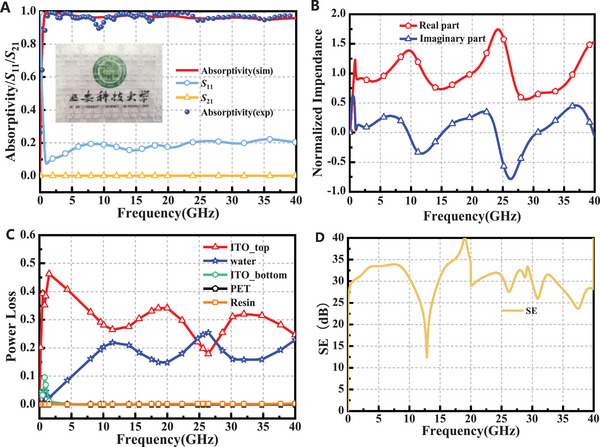
Results of the designed MA. (A) Absorptivity and *S*‐parameter of the designed water‐based absorber. (B) Normalized impedance. (C) Power loss of each component of the designed water‐based absorber. (D)Corresponding shielding effectiveness.

Figure 2a illustrates the absorption, reflection, and transmission spectra, of the designed absorber. Within the frequency range of 0.52–40 GHz, the absorber demonstrates perfect absorption, with absorption exceeding 90%. The relative bandwidth (RB) achieves a remarkable value of 194.9%. By employing the normalized impedance equation, and given that *S*_21_ equals zero, the equivalent impedance of the designed MA can be calculated by *Z_in_* = (1 + *S*_11_)/(1 − *S*_11_). Perfect absorption of incident EM waves occurs when the real part of the impedance equals 1 and the imaginary part equals 0. As shown in Figure 2b, the normalized impedance of the designed absorber aligns with this theoretical condition, ensuring effective broadband absorption performance. To elucidate the underlying absorption mechanism and to quantitatively deconvolve the contributions of different components, the power loss distribution of each material within the MA is simulated and is presented in Figure 2c. As clearly observed in Figure 2c, the patterned top ITO layer is unequivocally the primary contributor to microwave energy dissipation. Its power loss curve (red solid line) dominates across the entire 0.52–40 GHz spectrum. Specifically, in the low‐frequency region (below approximately 10 GHz), the power loss of the patterned top ITO layer peaks at values exceeding 0.45. This dominant loss at low frequencies confirms that the patterned ITO is the main driver for the fundamental electrical resonance and is responsible for the initial absorption band.

In contrast, the water exhibits a complementary yet crucial role. Its power loss (blue dashed line) is minimal at low frequencies but increases monotonically with frequency. It becomes substantial in the high‐frequency range (above 20 GHz), reaching a significant value of approximately 0.25 around 35–40 GHz. This frequency‐dependent behavior indicates that the water primarily functions as a dielectric loss medium, whose polarization relaxation mechanisms become increasingly effective at higher frequencies. Therefore, the water acts as a secondary but essential component that significantly enhances the absorption bandwidth and efficiency at high frequencies. The negligible power loss from the bottom ITO, PET substrate, and resin matrix confirms that they contribute minimally to the absorption, serving primarily as structural and functional layers for transparency.

Consequently, ultra‐broadband absorption performance is achieved through a synergistic mechanism: the patterned ITO provides the strong, resonant absorption at low frequencies, while the water filler extends the performance to higher frequencies via broadband dielectric loss. This clear division of roles resolves the conventional trade‐off between resonant strength and operational bandwidth, which is a key innovation of our hybrid design.

The shielding effectiveness (SE), which quantifies the attenuation of the electromagnetic wave by the material, was calculated from the measured S‐parameters. The total shielding effectiveness (SE) is defined as the ratio of the incident to transmitted power, expressed in decibels (dB):

The practical deployment of an electromagnetic interference (EMI) shield in a coal mine demands effective performance under oblique incidence, as electromagnetic (EM) waves undergo multiple reflections and scattering within the tunnel environment. Based on typical mine tunnel geometries and EM wave propagation studies, the dominant incident energy is expected to arrive within an angular range of up to 60° from the normal—this practical constraint underscores the necessity of evaluating absorber performance under non‐normal incidence, while also highlighting that polarization characteristics are a critical factor influencing absorber design.

To systematically assess the designed metamaterial absorber (MA), its polarization insensitivity was first evaluated under vertically incident transverse electric (TE) mode waves, with polarization angles ranging from 0° to 45°. The results confirm that the MA exhibits robust polarization insensitivity, as detailed in Section S3a. Another key aspect of performance evaluation is the absorber's response to oblique incidence; accordingly, absorption was simulated for oblique incidence angles spanning 0° to 60° (with a step size of 20°) in both TE and transverse magnetic (TM) modes. As elaborated in Section S4 (with additional details in Section S4b,c), the MA demonstrates excellent angular stability: for TM polarization, absorption remains above 90% even at 60° incidence across nearly the entire operational band; while TE polarization performance shows a gradual decline at higher angles, it still maintains over 80% absorption at 60° for a broad frequency range, consistent with the observation that oblique incidence absorption performance in TM mode surpasses that in TE mode.

The superior oblique incidence performance of the MA in TM mode can be attributed to the parallel alignment of the magnetic field direction with the MA surface, which effectively stimulates induced currents and enhances absorption stability. In contrast, the magnetic field direction in TE mode varies with the incidence angle, leading to a reduction in induced currents and a consequent decline in absorption performance. These findings not only underscore the importance of integrating polarization and incidence angle considerations into the optimization of absorber design but also confirm that the MA's performance aligns well with the practical angular distribution of EM waves in mine tunnels—validating its suitability for real‐world mining applications where non‐normal incidence is the rule rather than the exception.

### 2.2 Parametric Analysis of the Lowest Absorption Frequency

**FIGURE 3:**
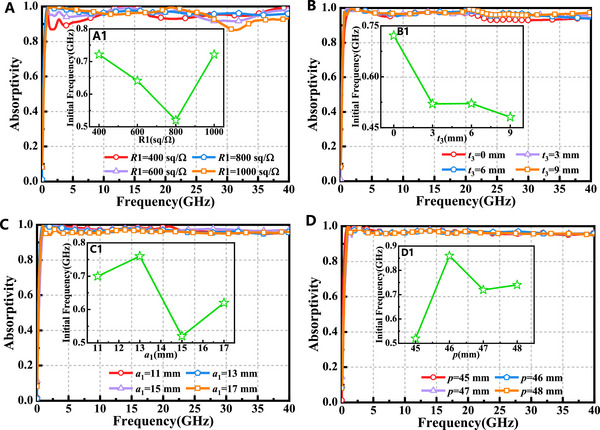
Influence of absorber structure parameters on absorption performance: (A)*R*1. (B)*t*_3_. (C)*a*_1_. (D)*p*.

To further investigate their influence, the impact of key parameters, including the ITO conductive film and water layer, on absorption performance was examined. Figure 3a illustrates the simulation results of the absorption for varying square resistances. As the square resistance *R*1 increases from 400 to 1000 Ω/sq in increments of 200 Ω/sq, the initial absorption frequency shifts to higher values, while a slight reduction in absorption is observed at higher frequencies. Figure 3b depicts the absorption characteristics as the water layer thickness *t*_3_ increases from 0 to 9 mm in steps of 3 mm. With increasing water layer thickness, absorption at higher frequencies improves, and the initial absorption frequency shifts to lower frequencies. To optimize the absorption bandwidth while minimizing the absorber size, the water layer thickness was set to 3 mm. Figure 3c illustrates the absorption as the width *a*_1_ of the central square ring in the resonant layer pattern increases from 11 to 17 mm in increments of 2 mm. As the width of the square ring expands, the absorption decreases, and the initial absorption frequency shifts to lower frequency. Based on the principle of maximizing absorption while minimizing the initial absorption frequency, the width of the square ring was set to 15 mm. In Figure 3d, the periodic unit size *p* was varied from 45 to 48 mm, revealing negligible changes in absorption performance. However, the initial absorption frequency exhibited a redshift. To ensure minimal absorber size and reduce fabrication costs, the periodic side length p was set to 45 mm. These findings highlight the importance of parameter optimization in achieving desired absorption performance while maintaining practical design considerations.

### 2.3 Wide‐Band Absorption Mechanism of the Designed Water‐Based MA

To gain deeper insight into the evolution of energy dissipation and transfer between the ITO and water layers as a function of frequency, we analyzed the power loss density distributions at several representative frequencies (Figure S4). At low frequencies (e.g., 1.07 GHz), the power loss is predominantly localized at specific resonant features of the top patterned ITO layer, such as the edges of the square rings. This suggests that ohmic loss within the ITO is the dominant dissipation mechanism at this stage, where strong localized electrical resonances effectively convert incident electromagnetic energy into heat. As the frequency increases to the mid‐range (e.g., 14.54 GHz), the region of power loss expands across the ITO layer, while significant loss begins to develop and intensify within the water layer. This behavior reflects the increasing role of dielectric loss caused by polarization relaxation of water molecules. At higher frequencies (e.g., 30.92 GHz), the power loss density in the water layer becomes substantially more pronounced, even exceeding that in the ITO layer in certain areas. This progression clearly validates the proposed synergistic dissipation mechanism: the patterned ITO layer initially drives low‐frequency resonance and dominates energy dissipation at lower frequencies, whereas the water layer serves as a broadband dielectric loss medium, with its contribution becoming critical for sustaining high absorption performance at higher frequencies. The observed frequency‐dependent spatial redistribution of energy dissipation is essential for achieving perfect absorption across the ultra‐wideband spectrum.

A comparative analysis between the proposed absorber and several similar absorbers is presented in Table 1. The results demonstrate that the proposed absorber exhibits a broader operating bandwidth, superior optical transparency, and a lower initial operating frequency compared to existing designs. Furthermore, its reduced structural thickness highlights significant potential for applications in electromagnetic compatibility (EMC) for compact electronic devices. These advantages underscore the proposed absorber's enhanced performance and practical applicability in advanced electromagnetic wave management.

**TABLE 1:** Comparison of the proposed absorber with previously reported water‐based MA.

Ref.	90% absorption band [GHz]	Relative bandwidth [%]	Optical transparency	Electric size^a^	Thickness (mm)
[31]	8.3–15.2	58.7	No	0.13λ_L_	∼5.0
[33]	12.49–98.21	154.9	Yes	0.15λ_L_	∼6.5
[38]	8.1–22.9	95.5	No	0.15λ_L_	∼5.5
[39]	9.6–98.9	165	No	0.10λ_L_	∼3.2
[41]	7.6–12.5/12.8–18.2	55	Yes	0.10λ_L_	∼4.0
[42]	9.44−120.92	171	Yes	0.09λ_L_	∼2.8
This work	0.52–40	194.9	Yes	0.02λ_L_	13.0

^a)^ The electric size is defined as the ratio of the practical thickness and wavelength at low frequency λ_L_.

### 2.4 Assessment of Electromagnetic Shielding Effectiveness for the Designed MA

In the underground coal mine environment, the operation of large electrical equipment, such as mining machines, frequency converters, and wireless base stations, generates a complex EM field, as illustrated in S7. This intricate electromagnetic environment presents significant challenges for the numerous sensitive electronic devices and instrumentation used in mines, including air pressure gauges and electronic display screens [49, 50]. Such devices are highly vulnerable to EMI, which can result in inaccurate readings and potentially lead to hazardous operational errors. Consequently, it is essential to implement effective electromagnetic shielding solutions for these electronic devices while maintaining high optical transparency to ensure that critical readings remain visible and can be promptly and accurately monitored. This dual requirement underscores the need for the development of advanced shielding materials capable of providing robust EMI protection without compromising the visibility of essential instrument displays.

#### 2.4.1 Anti‐Electromagnetic Interference Experiment

**FIGURE 4:**
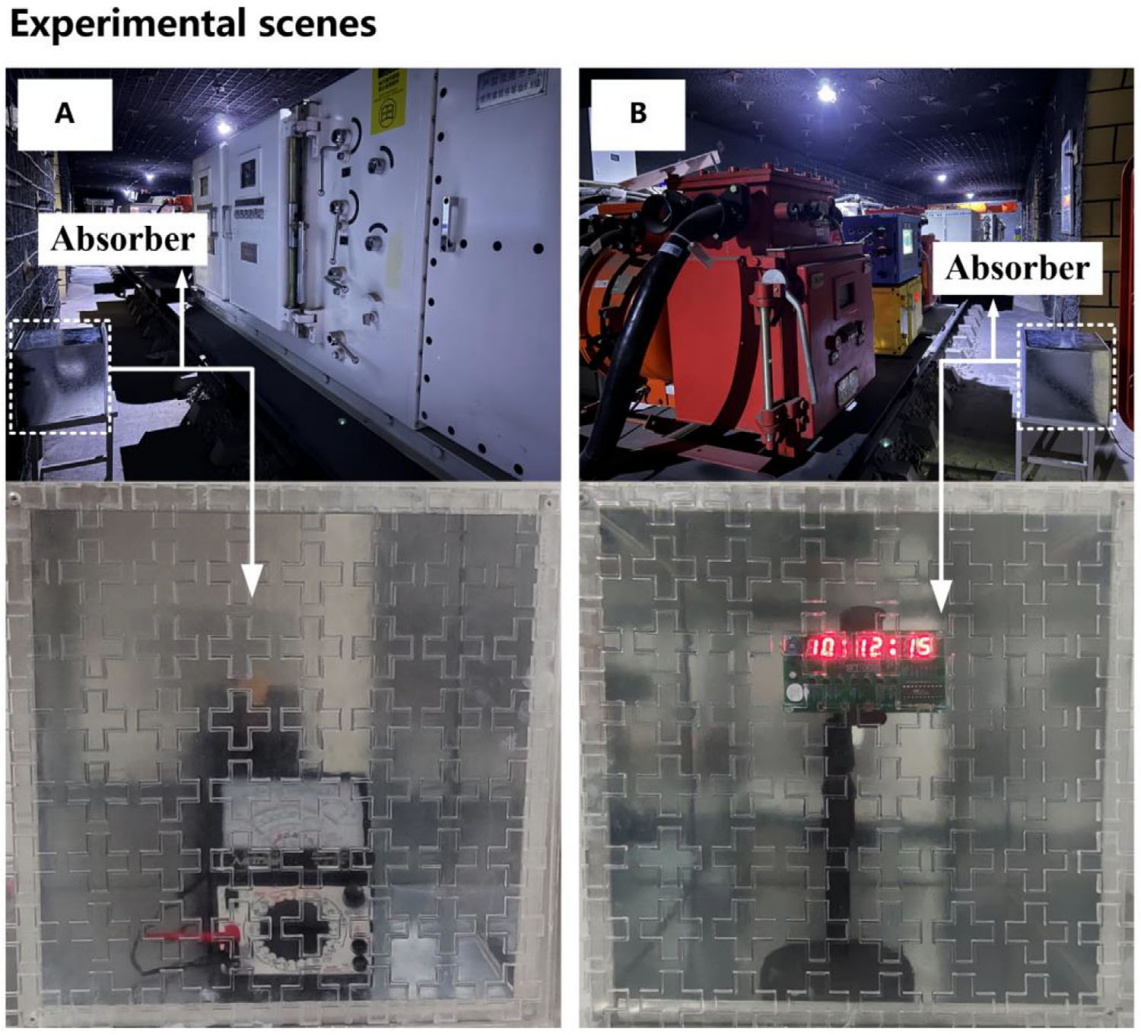
EM protection experiment: (A) Pointer multimeter. (B) Electronic clock based on single chip microcomputer.

In this study, two separate experiments were conducted within a simulated mine environment at the Comprehensive Experimental and Training Center for Coal Subject Specialty, Xi'an University of Science and Technology, to evaluate the EM protection efficacy of a designed absorber. The first experiment examined the EM shielding performance of the absorber on analog meters under complex electromagnetic conditions, as illustrated in Figure 4a. The second experiment employed a microcontroller‐based digital electronic clock to further demonstrate the absorber's EM protection capabilities, as shown in Figure 4b. To replicate a high‐intensity EMI environment, the experiments were carried out in a simulated tunnel. Electrical equipment, including frequency converters, was utilized to generate high‐power EMI signals capable of disrupting sensitive electronic devices. Analog multimeters and electronic clocks were placed inside an uncovered metal enclosure and positioned in proximity to the high‐power electrical equipment. The display status of the internal electronic components was monitored both with and without the absorber to evaluate its shielding effectiveness.

**FIGURE 5:**
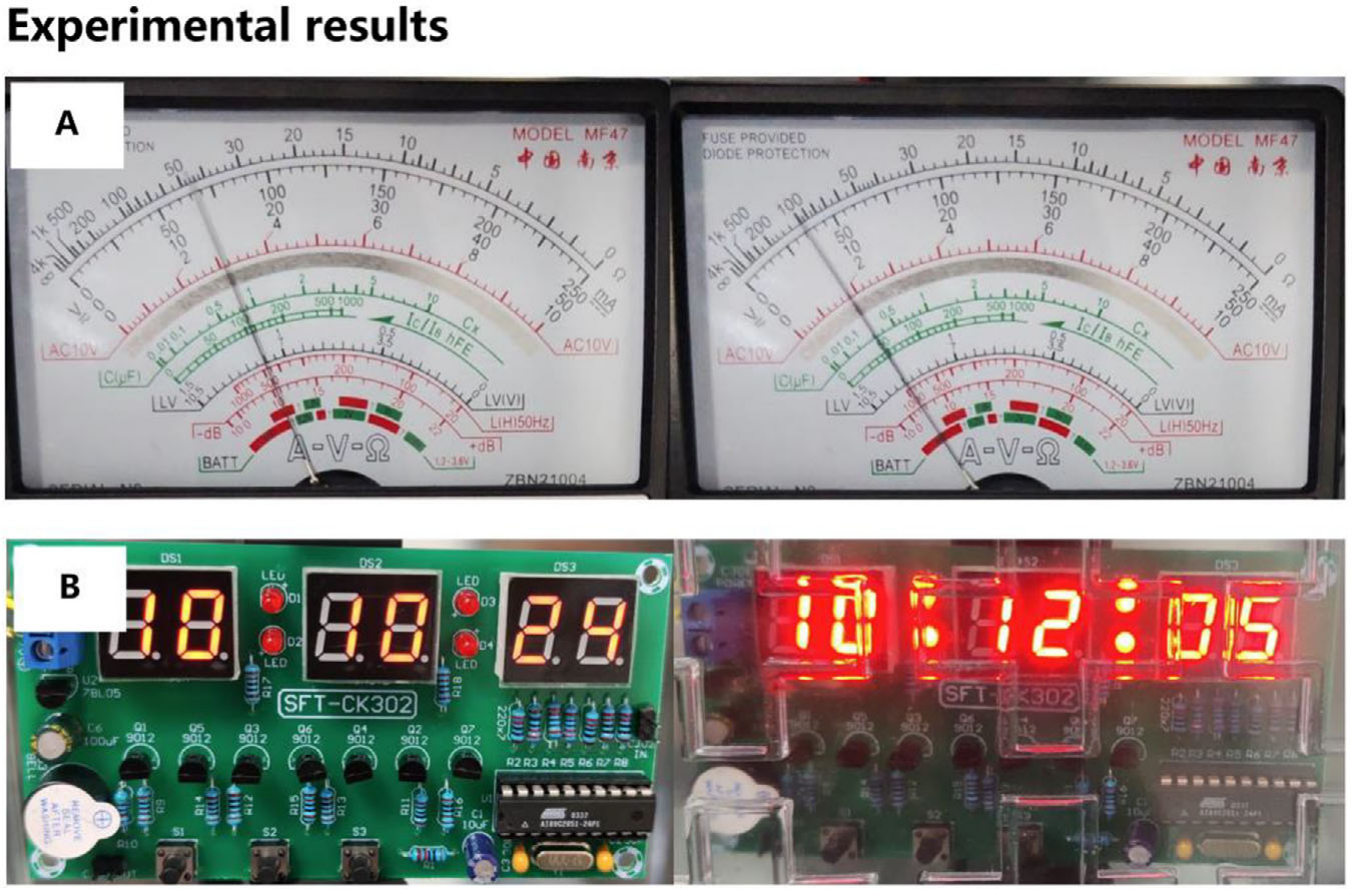
Electromagnetic protection experiments results: (A) Pointer multimeter with EMI (B) Electronic clock with/without absorber under EMI.

The experimental results are presented in Figure 5. In the first experiment, a pointer‐type analog multimeter was employed to measure the voltage across an electronic component. As depicted in Figure 5a, when measuring a 100‐ohm resistor on a circuit board, significant EMI caused the multimeter readings to fluctuate between 10 and 90 ohms, introducing substantial measurement errors (see Video S1 for detailed experimental results). This approach enabled the assessment of EMI's impact on voltage measurements and the effectiveness of the absorber in mitigating such interference. In the second experiment, the electronic clock was initially exposed to EMI without the absorber. Under these conditions, the digital display of the clock exhibited flickering, and the time display became inaccurate, as shown in Figure 5b (see Video S2 for detailed experimental results). Subsequently, the absorber was applied to the top layer of the open metal box, and the same EMI conditions were reintroduced. By observing the circuit board's display during the experiment, the absorber's ability to counteract electromagnetic interference was evaluated.

By comparing the responses of both the digital clock and the analog multimeter in the presence and absence of the absorber, the effectiveness of the absorber in shielding electronic devices from electromagnetic disturbances was systematically assessed, thereby validating its potential for electromagnetic protection applications. Additionally, a signal shielding experiment was conducted to further evaluate the absorber's performance. The results demonstrated that the designed absorber could completely block Wi‐Fi signals and significantly attenuate mobile communication signals (see Section S8, for further details [51]).

### 2.5 Discussion on Power‐Dependent Performance and Operational Boundaries

While the proposed MA demonstrates exceptional performance under standard conditions, its behavior under varying power levels warrants discussion to define operational boundaries. The electromagnetic absorption mechanism in our MA relies on two fundamental loss processes: ohmic loss in the patterned ITO layer and dielectric relaxation loss in the water medium. Both mechanisms are inherently linear at the electric field strengths typically encountered in coal mine EMI environments [47, 48]. The ITO film operates in its linear resistive region, while water's dielectric response follows the Debye relaxation model, which remains linear for field strengths below the threshold for electrochemical effects. This theoretical foundation indicates that the absorption efficiency and shielding performance of our MA should remain stable and independent of incident power under normal operating conditions.

The primary limitation for high‐power operation is therefore thermal rather than electromagnetic. The maximum sustainable incident power is determined by the structure's ability to dissipate heat without undergoing functional degradation. Potential failure mechanisms include water evaporation, deformation of the PET substrate, or damage to the ITO films. Based on the thermal properties of the constituent materials and typical EMI field strengths measured in mining environments [1, 2], the proposed MA possesses adequate thermal margin for its intended application. Consequently, our absorber is expected to maintain stable ultra‐broadband performance under all realistic operating conditions in coal mines, affirming its suitability for challenging industrial settings.

## 3 Conclusion

In conclusion, we propose a miniaturized metamaterial absorber (MA) based on the indium tin oxide (ITO)‐water synergistic loss mechanism, which employs optically transparent ITO conductive films and a water‐filled resin substrate to achieve ultra‐broadband electromagnetic (EM) wave absorption and structural transparency. The proposed MA exhibits three core performance advantages: ultra‐wideband absorption, high optical transmittance, and a low initial absorption frequency. Specifically, it realizes over 90% absorption in the 0.52–40 GHz frequency range, with an exceptional 194.9% relative absorption bandwidth, while maintaining a compact electrical size of merely 1/50 of the maximum operating wavelength. Quantitative assessments based on S‐parameters demonstrate that the proposed MA achieves a total Shielding Effectiveness (SE_t_) of over 10 dB across the entire 0.52–40 GHz ultra‐wideband, with SE_t_ frequently exceeding 25 dB at most frequencies. These quantitative results conclusively validate the MA's capability to safeguard sensitive electronic equipment while ensuring unobstructed visual monitoring—attributed to its inherent high optical transparency. Compared with previously reported MAs, the proposed design exhibits superior comprehensive performance, characterized by a wider relative absorption bandwidth, reduced thickness, and a lower initial absorption frequency. These prominent attributes highlight its significant application potential in EM shielding scenarios, particularly for providing effective EM protection and visual accessibility for sensitive electronic components operating in complex EM environments.

## 4 Experimental Section

### 4.1 Numerical Simulation

The simulation of the metamaterial absorber was performed using CST Studio Suite 2021 for both model construction and analysis. Periodic boundary conditions were applied to the unit cells along the x and y dimensions, while the *z* ‐axis was configured with an open boundary to replicate free‐space conditions. Electromagnetic wave incidence was simulated along the negative *z* ‐direction. To achieve optimal absorption performance, a parametric sweep was conducted within the software to systematically refine the design parameters. In the simulation, the complex permittivity was specified as 4.0(1 ‐ j0.001) for the resin and 3.1(1 ‐ j0.03) for the polyethylene terephthalate (PET) substrate. The low‐square‐resistance indium tin oxide (ITO) layer was characterized by a sheet resistance of 8 Ω/sq. The resistive value of the ITO layer within the resonant structure was optimized through a parametric study, with the detailed results provided in S3.

### 4.2 Sample Fabrication

An 8 × 8 array resin shell was fabricated using 3D printing technology, with overall dimensions of 364 × 364 × 13 mm^3^. To ensure watertight integrity, the periphery of the absorber was enclosed with a 4 mm resin frame, which included two small apertures to facilitate water circulation. Conductive films with two distinct sheet resistances were applied to the upper and lower surfaces of the resin shell, after which the structure was filled with deionized water (conductivity <5 µs/cm at 25°C). The dielectric constant of the processed transparent resin was confirmed to be 4.0(1 ‐ j0.001), closely aligning with the value used in the simulation. The low sheet resistance indium tin oxide (ITO) conductive films had a thickness of approximately 185 nm, while the high sheet resistance ITO films were approximately 5 nm thick. Both polyethylene terephthalate (PET) substrate layers were uniformly 0.188 mm in thickness, with a relative permittivity of 3.1(1 ‐ j0.03), consistent with the material specifications utilized in the simulations.

### 4.3 Microwave Measurement

The microwave measurements of the fabricated MA samples were conducted using the free‐space method. Three pairs of broadband horn antennas, each covering distinct frequency bands (0.3–2, 2–18, 18–40 GHz), were employed, with one antenna serving as the transmitter and the other as the receiver. These antennas were connected to the input and output ports of a vector network analyzer (VNA, ROHDE&SCHWARZ) via transmission lines. Prior to measuring the sample, the antennas were calibrated for each frequency band using a metal plate of identical dimensions to the MA sample as the reference. The measured reflection coefficient (*S*∼11∼) of the sample was then normalized against this reference to ensure accuracy. Both antennas were positioned on the same side of the MA sample, and the VNA was configured to emit electromagnetic waves at the corresponding frequency bands. This setup was used to measure the reflection coefficient (*S*∼11∼) of the sample, which was used to calculate the absorption.

### 4.4 Experimental Environment

The absorption tests were conducted in a microwave anechoic chamber, and the experiment details are presented in Figure S5. The measured reflection coefficient of the sample was used to calculate the absorption, thereby verifying the consistency between the experimental and simulated results. Further details of the experimental setup are provided in S5. The Electromagnetic protection performance tests were carried out in a simulated mine in the Comprehensive Experimental and Training Center of coal subject Specialty, Xi'an University of Science and Technology.″

## Conflicts of Interest

The authors declare no conflicts of interest.

## Supporting information




**Supporting File 1**: advs73785‐sup‐0001‐SuppMat.docx.

**Supporting File 2**: advs73785‐sup‐0002‐video1.mp4.

**Supporting File 3**: advs73785‐sup‐0003‐video2.mp4.

## Data Availability

The data that support the findings of this study are available from the corresponding author upon reasonable request.
