# Supplementary material for: An Optically Transparent Water‐Based Metamaterial Absorber for Ultra‐Broadband EMI Shielding in Coal Mines
Source: Adv Sci (Weinh). 2026 Jan 18;13(17):e18619. doi: 10.1002/advs.202518619 (PMC13042851; doi:10.1002/advs.202518619)
Supplement: Supplementary file 1 — Supporting File 1: advs73785‐sup‐0001‐SuppMat.docx. [file ADVS-13-e18619-s002.docx]

Supporting Information

An Ultra-Wideband Optically Transparent Water-Based Metamaterial Absorber for Robust Electromagnetic Shielding

*Xiaojun Huang*, *Lina Gao, Yifei Wang, Wei Hou*

**1. Dielectric constant of ITO conductive film**

Indium-Tin Oxide (ITO) conductive films are advanced transparent conductive coatings, produced by depositing a layer of indium tin oxide onto transparent organic film substrates through sputtering, complemented by a high-temperature annealing treatment. [1]

The Drude-Lorentz model stands as a quintessential representation of the free electron gas theory, adept at capturing the essence of electron dynamics within metallic structures, which in turn dictates the material's electrical and thermal properties.

When ITO serves as a transparent conductive film, its electromagnetic properties can be estimated using the parameters of the Drude model, with the expression given by:[2]

$\varepsilon_{r}\left(\omega\right)=\varepsilon_{\infty}-\frac{\omega_{p}^{2}}{\omega\left(\omega+i\omega_{c}\right)}$ (S1)

Among it, $\varepsilon_{\infty}=3.9$, plasma resonance frequency$\omega_{p}=461$ THz, damping frequency$\omega_{c}=28.7$ THz, and incident wave frequency is $\omega$. Therefore, it can be concluded that the complex dielectric constant of ITO in the research frequency range (0 GHz -40 GHz) is shown in **Figure S1**. The real part is about -254, which means it has a large refractive index, reflecting its metal like properties and can achieve strong absorption of incident EM waves.

**Figure S1.** The permittivity of ITO in the investigated frequency band.

**2. Dielectric constant of water**

Water's elevated dielectric constant bestows upon it a pronounced capacity to modulate the propagation of electromagnetic waves, offering a robust loss mechanism for metamaterial absorbers. This mechanism is instrumental in augmenting the absorption and dissipation of electromagnetic energy. Moreover, the dielectric loss angle tangent of the water medium, indicative of its loss angle, plays an equally pivotal role in the loss characteristics of these absorbers. It reflects the medium's proficiency in transforming electromagnetic energy into heat, a key attribute for realizing high-efficiency absorption. The intrinsic properties of water enable metamaterial absorbers to demonstrate intense energy dissipation across a defined frequency spectrum, ensuring effective electromagnetic wave absorption. The complex permittivity of water is determined through the Debye equation [3]:

$\varepsilon(\omega,T_{\text{water}})=\varepsilon_{\infty}(T_{\text{water}})+\frac{\varepsilon_{s}(T_{\text{water}})-\varepsilon_{\infty}(T_{\text{water}})}{1-i\omega\tau(T_{\text{water}})}$ (S2)

Where, $\varepsilon_{s}$、$\varepsilon_{\infty}$and $\tau$are the static permittivity, optical permittivity, and rotational relaxation time, respectively. These parameters are all temperature-dependent:

$\varepsilon_{s}(T_{\text{water}})=a_{1}-b_{1}T_{\text{water}}+c_{1}T_{\text{water}}^{2}-d_{1}T_{\text{water}}^{3}$ (S3)

$\varepsilon_{\infty}(T_{\text{water}})=\varepsilon_{s}(T_{\text{water}})-a_{2}e^{-b_{2}T_{\text{water}}}$ (S4)

$\tau(T_{\text{water}})=c_{2}e^{T_{1}/(T_{\text{water}}+T_{0})}$ (S5)

Where, a_1_=87.9, b_1_=0.404K−1, c_1_=9.59×10−4 K−2, d_1_=1.33×10−6 K−3, a_2_=80.7, b_2_=4.42×10−3 K−1, c_2_=1.37×10−13 s, T_0_=133°C, T_1_=651°C。Utilizing the provided equation, **Figure S2** illustrates the dielectric constant curve of water concerning frequency within the temperature range of 0°C to 100°C. For this investigation, the liquid medium employed is pure water at room temperature (23°C).

**Figure S2.** Permittivity of water at different temperatures.

**3. Polarization and oblique incidence characteristics of water-based absorber**

The design of an absorber is significantly influenced by its polarization attributes. In **Figure S3**a, absorption is evaluated under 0°-45° polarization angles with vertically incident TE mode. The symmetrical geometry of the absorber with respect to both the x and y axes, the absorption remains consistent across the various polarization angles, demonstrating the robust polarization insensitivity about the designed MA. Oblique incidence is another crucial aspect to consider in absorber assessment. In Figure S4b,c, absorption is simulated for oblique incidence angles from 0° to 60° in both TE and TM modes, with a 20° step size. The absorption gradually decreases with the increase in incidence angle, and the impact on absorption performance is more pronounced in TE mode than TM mode. In TE mode, with an incident angle of 40°, the absorption remains above 80%; while in TM mode, it stays above 90%. Even at the incidence angle of 60°, the absorption in TE mode remains above 75%, while in TM mode, it remains above 80%. The superior oblique incidence performance in TM mode can be attributed to the parallel orientation of the magnetic field direction to the MA surface, stimulating induced current and enhancing absorption stability. In contrast, the changing magnetic field direction of TE mode with the incidence angle results in a decrease in induced current and, consequently, a reduction in absorption performance.


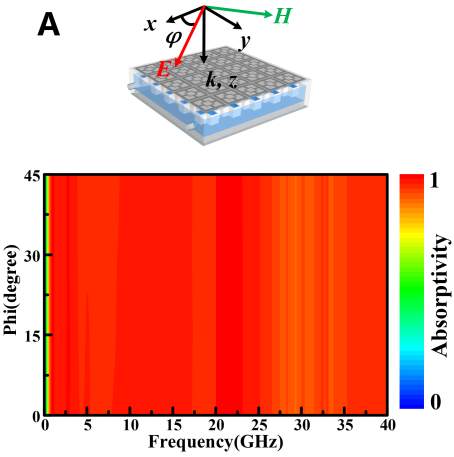

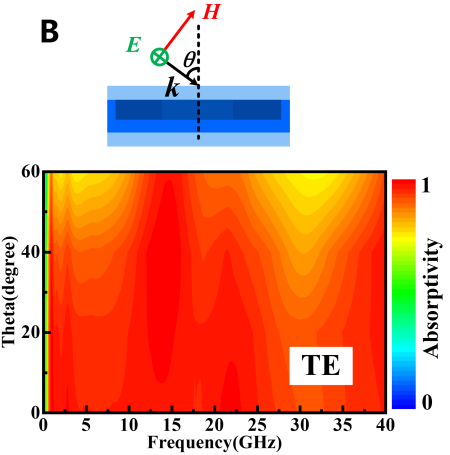

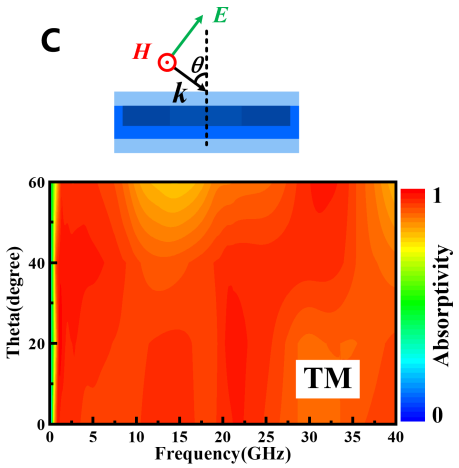


**Figure S3.** (a) The absorptivity corresponding to polarization angles from 0° to 45. The absorptivity corresponding to incident angles from 0° to 60° (b) TE mode. (c) TM mode

4. Electromagnetic field analysis of the designed water-based MA

Field monitors are strategically positioned at various resonant points to elucidate the wide-band absorption mechanism of the designed MA. This analysis involves examining the surface current, electric field, magnetic field, and power loss density at resonant points. Figure S4a, depicts the distribution of top and bottom surface currents at four resonant points. The top current concentrates mainly in the ITO film distribution, driven by the directional movement of charge carriers when EM waves induce current on the conductive film surface. This phenomenon results in EM resonance at various frequencies, facilitating broadband absorption. At 1.07 GHz and 30.92 GHz, the top resonant layer generates an induced current, antiparallel to the reflective backplane, forming a closed loop in the *z*-axis, thereby generating magnetic resonance under electric field excitation. Conversely, at 14.54 GHz and 20.6 GHz, the induced current between the top resonant layer and the backplane is in the same direction, resulting in electric resonance.

Subsequently, the wide-band absorption mechanism of the absorber is further explained in terms of electric field and magnetic field distribution and power loss density. Figure S4b shows the electric field distribution (*x*=0) and the power loss density (bottom) at the cross section of the resonant point, respectively. The resulting ring electric field is indicated by a curved black arrow, which can be attributed to dielectric relaxation. Water molecules with high permittivity in the absorbers are polarized, acting as electric dipoles that need time to align with alternating magnetic field. When the frequency of the alternating field increases, the dipoles exhibit inertia (or time delay) in their response, leading to increased power losses. At 1.07 GHz and 14.54 GHz, the electric field is mainly distributed in the surface conductive film and the top layer of resin, and a small amount of electric field is also distributed in the water layer. However, at 20.6 GHz and 30.92 GHz, the electric field is mainly distributed in the conductive film and the top layer of resin, and there is almost no electric field distribution in the other parts. This further indicates that the electric resonance is mainly caused by the top conductive film.

Figure S4c illustrates the magnetic field distribution (*y*=0) and the corresponding power loss density at the cross-section of the resonant point. Similarly to Figure S4b, except that at 1.07 GHz, the magnetic field is concentrated at the bottom conductive film. This is because at 1.07 GHz, the top and bottom surface currents are in opposite directions, thus forming a closed loop in the z axis and generating magnetic resonance. Therefore, strong electric resonance and strong magnetic resonance exist at this frequency point simultaneously. The coexistence of electric resonance and magnetic resonance at each resonant point enables the designed MA to match the free-space impedance over a wide frequency range, achieving complete absorption of the incident EM waves. The dissipation of EM waves energy through the dielectric loss of water and the induced currents in ITO ultimately leads to perfect absorption.

The power loss density reveals that water molecules, being polar molecules with a high dielectric constant, are easily polarized in an electric field. When a high-frequency EM wave passes through water, the water molecules try to rotate with the rapid changes in the EM waves, thus consuming the energy of the EM waves and converting it into heat. As the frequency increases, the power loss density of the water layer gradually decreases due to the shallow penetration depth of high-frequency EM waves and the increasing dielectric loss factor of water. The incident EM waves are almost entirely absorbed before reaching the bottom of the water layer.


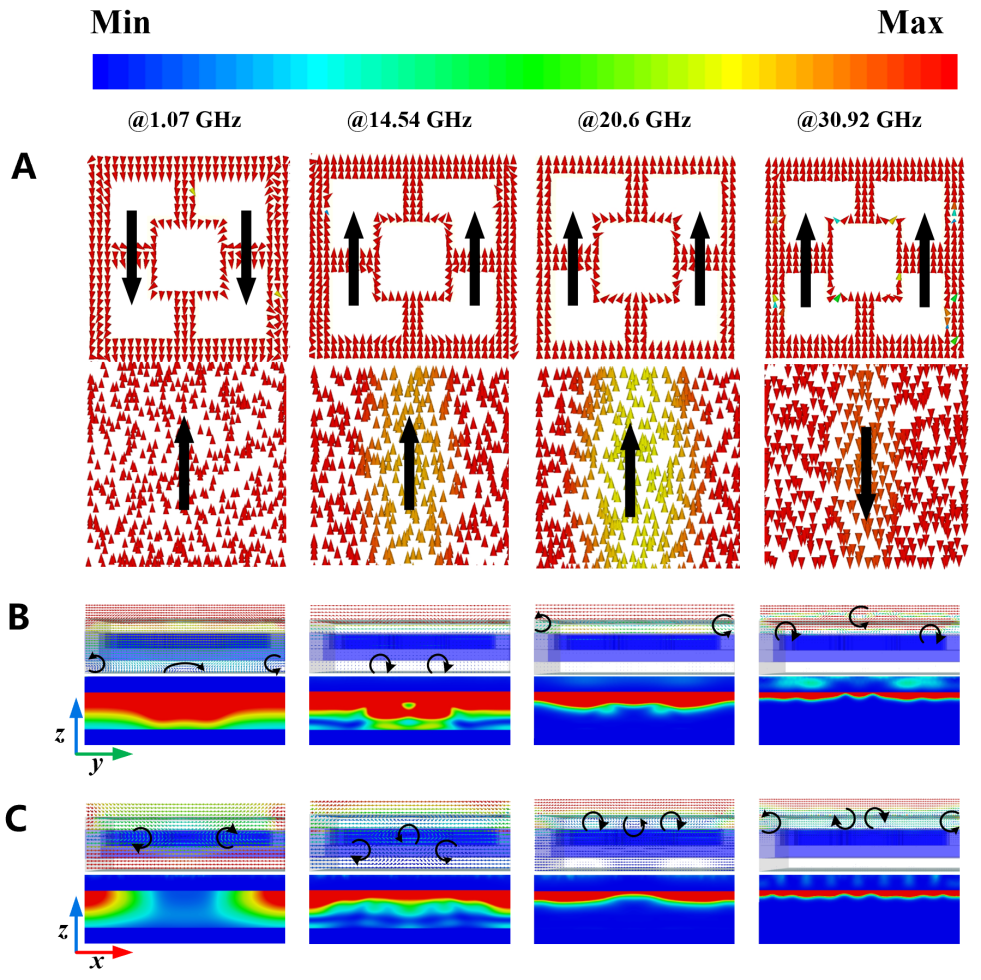


**Figure S4**. Field distribution at the resonant points of the designed MA. (a) Surface current distribution of the top and bottom layers of the resonant points. (b) Electric field (top) and power loss density (bottom). (c) Magnetic field (top) and power loss density (bottom).

**5. Experimental setup to verify MA absorption**


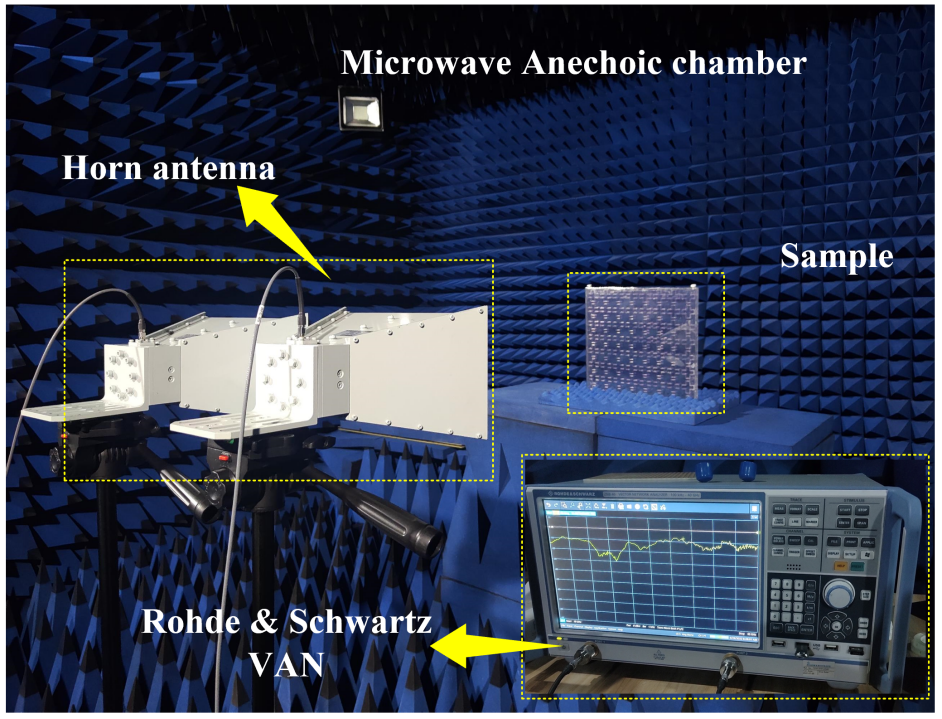


**Figure S5.** Experimental test environment and equipment.

**6. Tunability of the designed water-based absorber**

Simulations of the absorption are conducted for varying water layer thicknesses, specifically at 0mm, 1mm, 3mm, 5mm, and 7mm, achieved by selectively removing water through peripheral orifices, with the results depicted in **Figure S6**a. When the water is completely discharged, the overall absorption decreases, especially in 15.8-21 GHZ the absorption is below 80%, and the lowest absorption only about 67%. Two absorption bands are generated, with absorption greater than 90% at 1.3-14.9GHz and 21.7-40 GHZ. Figure S6b presents the simulation results of the absorption with water temperature adjustment. The dielectric constant of water, which is described by the Debye equation, varies with temperature, thus different water temperatures may lead to varying electromagnetic responses. The results show that the water temperature has little effect on the absorptivity of the absorber.

Adding different solutes, such as ethanol, NaCl, sugar, or other polar solutions, to water can alter the dielectric constant of water, thereby yielding distinct electromagnetic responses. The following simulation investigates the absorption of water media replaced by ethanol solutions of varying concentrations and NaCl solutions of different concentrations. The dielectric constant of ethanol can be calculated using the Debye equation [4], as shown in Figure S6c, with the corresponding absorption depicted in Figure S6d. Similarly, the dielectric constant of NaCl solutions can be determined using a modified Debye equation [5], with the corresponding absorption illustrated in Figure S6e.

**Figure S6.** (a) Absorptivity of different water layer thicknesses. (b) Absorptivity of different water temperatures. (c) Permittivity of ethanol at different concentrations. (d) Absorptivity of ethanol at different concentrations. (e) Absorptivity of NaCl solutions with different concentrations.

**7. Complex electromagnetic environment in coal mine**


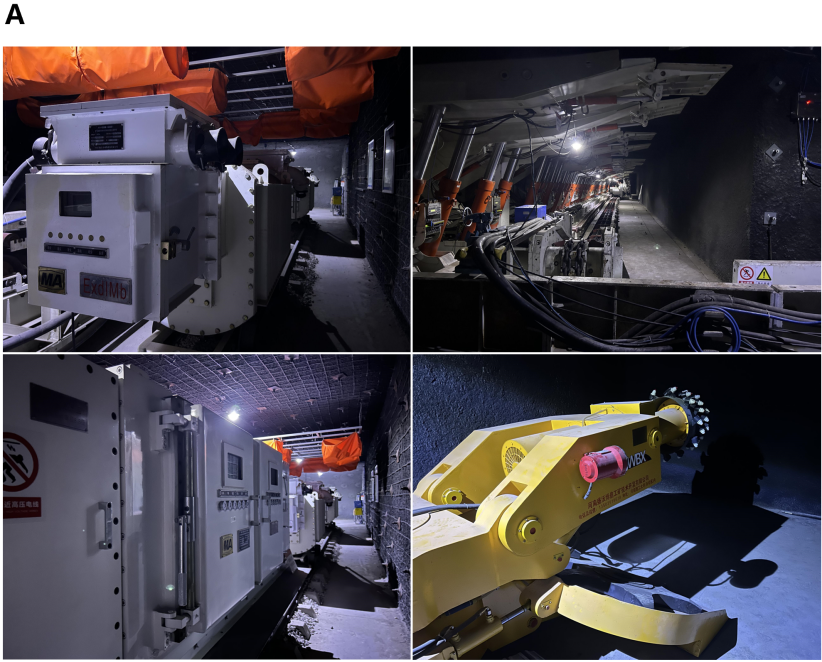


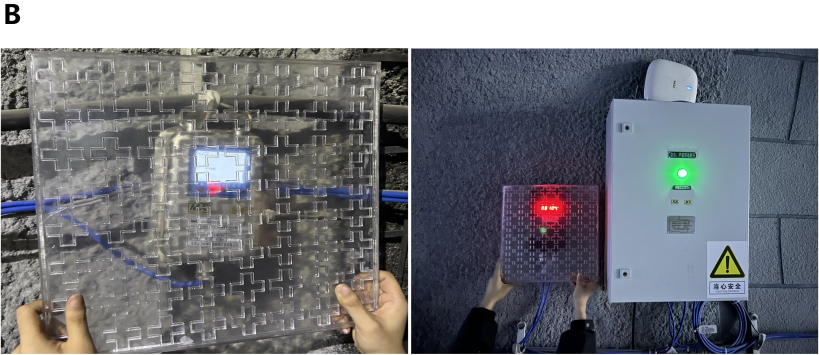


**Figure S7.** (a) Complex high-power EM environment. (b) High visibility

**8. Applied experiment of signal shielding**

The schematic diagram of signal shielding experiment setup is shown in **Figure S8a**. The experimental setup involved using a router to emit a Wi-Fi signal within the frequency range of 2.4 to 5.8 GHz, as well as a mobile signal from a nearby China Mobile base station in the 1.8 to 2.4 GHz range. To assess the impact of signal absorbers on shielding effectiveness, a cell phone was placed inside an open metal box. The signal strength was then measured both with and without the absorber in place. The effectiveness of the absorber on signal shielding was evaluated by observing the changes in signal strength before and after the absorber is introduced, as shown in the actual experimental environment. The entire experiment was carried out in free space to ensure sufficient signal strength and wide signal distribution. The model of cell phone used in the experiment is Mi 12, and the signal testing software is Cellular-Z. Experimental results are shown in **Figure S8b**. When connecting the router, the Wi-Fi signal strength is -55 dBm, the Wi-Fi signal completely disappears after placing the absorber, and the mobile phone shows no network connection; when receiving a mobile signal from a nearby base station, the signal strength decays from -57 dBm to -150 dBm with the usage the absorber. The complete blocking of Wi-Fi signals may be due to its fast transmission rate but weak penetration, while the penetration of mobile signals is strong, resulting in its strength attenuation but not completely blocked.


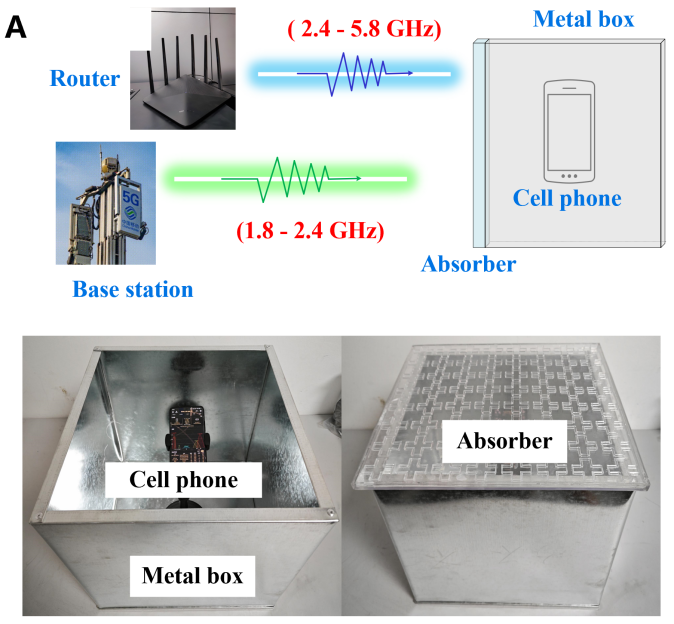

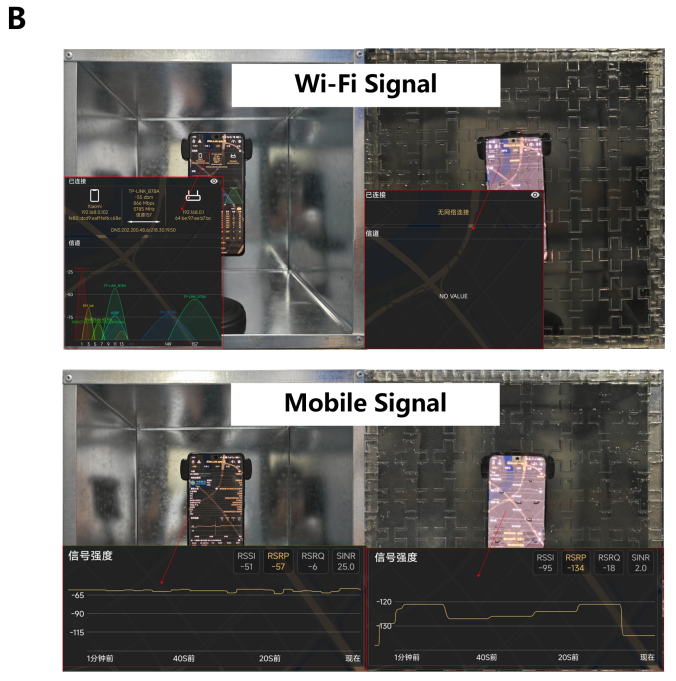


**Figure S8.** (a) Signal shielding experiment. (b) Electromagnetic shielding experiments results: Wi-Fi signal without and with absorber; Mobile signal without and with absorber.

**9: Discussion on Mechanical Robustness and Thermal Stability**

This section provides a discussion on the design considerations ensuring the mechanical and thermal stability of the proposed metamaterial absorber for deployment in coal mine environments.

S9.1 Mechanical Robustness
The structural design of the absorber prioritizes mechanical robustness. The use of a 3D-printed, high-strength photopolymer resin for the shell provides a monolithic and impact-resistant framework. The critical sealing against water leakage is achieved by bonding the flexible yet durable ITO-coated PET films to the top and bottom of the resin shell, creating a fully encapsulated and hermetic unit. This design is intrinsically capable of resisting the static loads and vibrational disturbances commonly present in operational mine tunnels, ensuring long-term structural integrity and functional performance without failure or leakage.

S9.2 Thermal Stability
The operational electromagnetic performance of the absorber is analyzed considering the temperature fluctuations in underground coal mines. The complex permittivity of water, the key lossy medium, follows the Debye relaxation model, which is a well-understood function of temperature. The typical ambient temperature range in coal mine roadways is sufficiently narrow (approximately 10°C to 30°C) such that the resulting shift in the absorption spectrum is minimal, particularly within the core operational band from 1 GHz to 40 GHz. The ultra-wideband characteristic of the absorber readily accommodates any minor low-frequency boundary shift, ensuring consistently high absorption performance. Furthermore, the ITO films and PET substrates are standard commercial materials known for their stable electronic and mechanical properties over this and even wider temperature ranges. Therefore, the design is deemed thermally robust for its intended application.

References

1. L. Álvarez-Fraga, F. Jiménez-Villacorta, J. Sánchez-Marcos 1, A. de Andrés, C. Prieto, *Appl. Surf. Sci.* **2015**, *344*, 217.
2. H. Xu, J. Cheng, Q. Huang, M. Luo, D. Li, D. Zhu, G. Zhan, Q. Zheng, Y. Zhang, J. Shao, C. Wu, *Opt. Lett*. **2024**, *49*, 89.
3. A. Andryieuski, S. M. Kuznetsova, S. V. Zhukovsky, Y. S. Kivshar, A. V. Lavrinenko, *Sci. Rep*. **2015**, *5*, 13535.
4. X. Kong, W. Lin, X. Wang, L. Xing, S. Jiang, L. Kong, M. Liu, *J. Opt. Soc. Am. B*. **2021**, *38*, 3277.
5. X. Yan, X. Kong, Q. Wang, L. Xing, F. Xue, Y. Xu, S. Jiang, X. Liu, *IEEE Trans. Antennas Propag.* **2020**, *68*, 6162.
